# “Rapid impact” 10 years after: The first “decade” (2006–2016) of integrated neglected tropical disease control

**DOI:** 10.1371/journal.pntd.0006137

**Published:** 2018-05-24

**Authors:** Peter J. Hotez, Alan Fenwick, Sarah E. Ray, Simon I. Hay, David H. Molyneux

**Affiliations:** 1 Texas Children’s Hospital Center for Vaccine Development, National School of Tropical Medicine, Baylor College of Medicine, Houston, Texas, United States of America; 2 Schistosomiasis Control Initiative, Imperial College London, London, United Kingdom; 3 Institute of Health Metrics and Evaluation, University of Washington, Seattle, Washington, United States of America; 4 Department of Parasitology, Liverpool School of Tropical Medicine, Liverpool, United Kingdom; London School of Hygiene and Tropical Medicine, UNITED KINGDOM

New data from the Global Burden of Disease (GBD) Study 2016 indicate substantial declines in the prevalence and disease burden (as measured in disability-adjusted life years [DALYs]) of six of the seven neglected tropical diseases (NTDs) targeted by integrated mass drug administration (MDA) over the previous decade.

## Introduction: The decade of NTDs

During the early 2000s, a concept was proposed for linking or integrating MDA in order to simultaneously target trachoma and six helminth infections—ascariasis, trichuriasis, hookworm, schistosomiasis, lymphatic filariasis (LF), and onchocerciasis [1, 2]. Central to the concept was that all seven of these infections were chronic and debilitating conditions and could be considered together as representing a subgroup of NTDs [1]. The seven NTDs targeted for MDA overlapped geographically and were often co-endemic (syndemic), so in many areas, individuals and populations living in poverty in Africa were polyparasitized [1–4].

For such African populations, a package of drugs, initially called “rapid impact,” could be delivered [1]. The drugs included either albendazole or mebendazole for ascariasis, hookworm, and trichuriasis; praziquantel for schistosomiasis; ivermectin and albendazole for LF (or in countries nonendemic for onchocerciasis, albendazole and diethylcarbamazine [DEC] for the nations of Comoros, Kenya, Madagascar, Zambia, and Zimbabwe); ivermectin for onchocerciasis; and azithromycin for trachoma [1, 2]. These medicines were donated by multinational pharmaceutical companies (originally Merck & Co. Inc., GlaxoSmithKline [GSK], Johnson&Johnson [J&J], Pfizer, and MedPharm, followed by others mentioned in the next section) or, in some cases, were available as low-cost generics. Moreover, there were several major nongovernmental development organizations (NGDOs) assisting African health ministries to implement vertical programs that could be modified to incorporate all seven diseases, thereby accelerating the elimination of LF, onchocerciasis, and trachoma, in addition to reducing morbidity for the three major soil-transmitted helminth infections and schistosomiasis [1].

**Fig 1:**
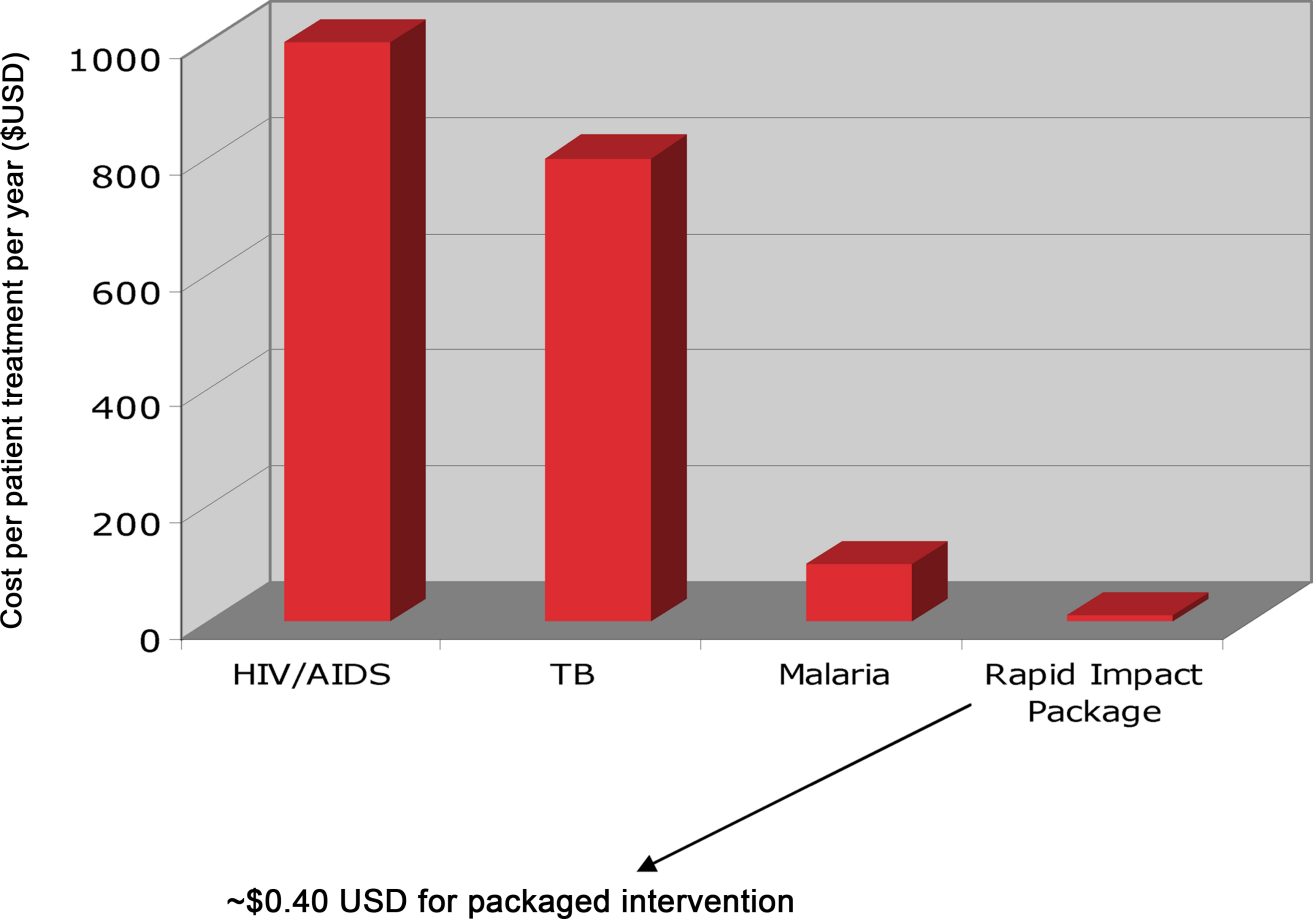
Range of treatment costs per year. Reproduced from [1].

It was further predicted that gains could be achieved in terms of efficiencies and cost-effectiveness. Indeed, because of the drug company donations, it was estimated that integrating NTD control through the rapid-impact package of essential medicines could reduce DALYs roughly approximating those of HIV/AIDS, tuberculosis, or malaria [4], but at the astonishingly and comparatively low cost of US$0.40 per person annually [1] (Fig 1). In some cases, the costs were even lower. This modest unit cost reflected several key cost-saving elements, including drug donations, economies of scale and synergistic deliveries, and a drug-distribution system that relied on volunteerism from community health workers and school teachers [5].

In addition, collateral benefits could also be achieved because of the impact of ivermectin on scabies and other ectoparasitic infections, as well as strongyloidiasis; albendazole on oesophagostomiasis; praziquantel on cestodes; and azithromycin’s ability to treat other bacterial infections, including yaws, and possibly adult mortality rates [1, 4, 6, 7, 8]. The rapid-impact package could eventually be incorporated in either HIV/AIDS or malaria control programs, especially where female genital schistosomiasis promoted horizontal AIDS transmission and hookworm exacerbated malaria anemia [4, 9].

## Progress since 2006

The concept of rapid impact helped to drive new pro-poor policies. By 2006, a new Department of NTDs was established at the World Health Organization (WHO) with Dr. Lorenzo Savioli as its inaugural director, and funds were appropriated by the United States Congress via USAID and the British Parliament through the UK Department for International Development to support implementation of integrated NTD control. Shortly thereafter, this approach expanded from Africa to Asia and the Americas. In 2012, the commitment of the major multinational pharmaceutical companies—which now also included donations for praziquantel (Merck KgaA); triclabendazole for fascioliasis (Novartis); and diethylcarbamazine citrate for LF (Eisai, LTD), among others (including additional commitments for human African trypanosomiasis, visceral leishmaniasis, and leprosy)—was reaffirmed through a London Declaration for NTDs in 2012. New NGDOs entered into the NTD space under umbrella organizations such as the Global Network for NTDs and Uniting to Combat for NTDs. These latter activities also reflected renewed interest by the Bill & Melinda Gates Foundation. The scale-up of MDA proceeded rapidly such that WHO reported that in 2016, an estimated 1.024 billion people had received at least one essential NTD medicine from the rapid-impact package, representing 62.3% of the world’s population requiring treatment [10].

The newest information released by the GBD Study 2016 reveals critical progress in reducing the global disease burden resulting from NTDs, both globally and within sub-Saharan Africa [11, 12]. Overall, the seven NTDs targeted for integrated MDA still rank among the prevalent infections of people living in poverty, with up to 800 million people living with ascariasis, 400 million with hookworm or trichuriasis, 190 million with schistosomiasis, 30 million with LF, 15 million with onchocerciasis, and 3 million with visual impairments from trachoma in the year 2016, but with substantial reductions in their global prevalence over the last decade. As shown in Table 1, between 2006 and 2016, there were between 13.5% and 45.3% reductions in the age-standardized disability-adjusted life years (DALYs) for each of the diseases globally, with the greatest impact for LF, ascariasis, schistosomiasis, and onchocerciasis [11, 12]. Similarly, in sub-Saharan Africa, more than 100 million people are infected with ascariasis, hookworm infection, trichuriasis, and schistosomiasis, but again, substantial reductions in age-standardized DALYs (between 13.4% and 41.1% reductions) were observed. Overall, the age-standardized DALYs for NTDs decreased 32% globally and 34% for sub-Saharan Africa.

**Table 1:** MDA treatment coverage and impact on prevalence and DALYs 2006–2016 (data from [11, 12]).

	Global				Sub-Saharan Africa			
Disease	Prevalence in 2016 [11]	All-age DALYs in 2016 [12]	Change in DALYs 2006–16 [12]	Change in age-standardized DALYs 2006–16 [12]	Prevalence in 2016	All-age DALYs in 2016	Change in DALYs 2006–16	Change in age-standardized DALYs 2006–16
Ascariasis	799.68 million	1.31 million	–31.2%	–37.0%	133.32 million	0.46 million	–22.8%	–38.0%
Hookworm	450.68 million	1.69 million	–4.2%	–13.5%	132.30 million	0.51 million	+14.0%	–13.4%
Trichuriasis	435.09 million	0.34 million	–20.0%	–27.8%	111.07 million	0.09 million	+2.2%	–21.4%
Schistosomiasis	189.77 million	1.86 million	–24.4%	–33.7%	162.29 million	1.58 million	–15.2%	–35.8%
Lymphatic Filariasis	29.38 million	1.19 million	–37.4%	–45.3%	14.34 million	0.67 million	–4.1%	–27.6%
Onchocerciasis	14.65 million	0.96 million	–24.0%	–32.6%	14.64 million	0.96 million	–23.9%	–41.1%
Trachoma	3.34 million	0.25 million	–0.5%	–23.8%	0.45 million	0.04 million	–20.5%	–39.1%
Total for 7 NTDs		7.6 million	–24%	–32%		4.31 million	–14%	–34%

**Abbreviations:** DALY, disability-adjusted life year; NTD, neglected tropical disease.

The reduction in DALYs likely reflects both the reduction in disease prevalence and intensity due to either the direct impact of MDA on worm burdens or, in some cases, to the interruption of disease transmission. In addition, there may be added effects due to reductions in disability that result from patient care. The data sources we report here do not enable a distinction between these pathways. Thus, while such information does not prove that MDA and other pro-poor policies produced these results, it presents a plausible hypothesis for future investigations given that there has been limited expansion of other determinants of infection such as specific health education, clean water provision, and improved sanitation.

It is also interesting to note that the least impact was for human hookworm infection—both globally and in sub-Saharan Africa, the age-standardized DALYs decreased only 13% [12]. This finding may reflect the observation that current MDA strategies mainly focus on children, whereas a high hookworm disease burden is also found among adults, as well as evidence for variable efficacies of single-dose mebendazole or albendazole [13].

For trachoma, the GBD prevalence estimates number of individuals living with visual impairments from blinding trachoma rather than those with active infection. As such, reductions in trachoma prevalence may directly reflect improvements in population health and access to eye care services but will not necessarily correlate with the impact of MDA over the last decade, which aims to reduce the transmission of infection, together with simple surgeries, face washing, and environmental control (known by the acronym of SAFE).

In addition, and as shown in Table 2, GBD 2016 reveals that annual declines in the DALYs from the seven NTDs accelerated during the 11 years between 2006 and 2016 and were greater than during the 27-year period from 1990 to 2016, both globally and specifically in sub-Saharan Africa. Notably, the average global annual declines in DALYs for schistosomiasis and LF accelerated roughly two-fold (94% and 116%, respectively) between 2006 and 2016 and 50% for onchocerciasis and 38% for trachoma, whereas the declines for ascariasis and trichuriasis were more modest—17% and 4%, respectively. Similar results were also achieved for sub-Saharan Africa, although overall the declines for intestinal helminth infections were more impressive than those achieved globally. The acceleration in declines in age-standardized DALYs rates within sub-Saharan Africa from 2006–2016 when compared to 1990–2016 were greatest for trichuriasis, schistosomiasis, and LF (1.9×, 1.8x, and 1.8×, respectively).

**Table 2:** Comparison of age-standardized reductions in DALYs from 2006–2016 compared to 1990–2006 (data derived from [1,2]).

	Global					Sub-Saharan Africa				
Disease	Change in age-standardized DALYs 1990–2016 [12]	Average percent decrease per year 1990–2016	Change in age-standardized DALYs 2006–2016 [12]	Average percent decrease per year 2006–2016	Acceleration between 2006–2016 vs 1990–2016	Change in age-standardized DALYs 1990–2016	Average percent decrease per year 1990–2016	Change in age-standardized DALYs 2006–2016	Average percent decrease per year 2006–2016	Acceleration between 2006–2016 vs 1990–2016
Ascariasis	–77.8%	–2.9% annually	–37.0%	–3.4% annually	1.2× (+17%)	–63.8%	–2.4% annually	–38.0%	–3.45% annually	1.4× (+44%)
Hookworm	–43.4%	–1.6% annually	–13.5%	–1.2% annually	None	–20.2%	–0.8% annually	–13.4%	–1.22% annually	1.5× (+53%)
Trichuriasis	–63.7%	–2.4% annually	–27.8%	–2.5% annually	1.0× (+4%)	–25.6%	–1.0% annually	–21.4%	–1.95% annually	1.9× (+95%)
Schistosomiasis	–41.9%	–1.6% annually	–33.7%	–3.1% annually	1.9× (+94%)	–47.3%	–1.8% annually	–35.8%	–3.25% annually	1.8× (+80%)
Lymphatic Filariasis	–51.5%	–1.9% annually	–45.3%	–4.1% annually	2.2× (+116%)	–36.8%	–1.4% annually	–27.6%	–2.51% annually	1.8× (+79%)
Onchocerciasis	–54.6%	–2.0% annually	–32.6%	–3.0% annually	1.5× (+50%)	–66.1%	–2.5% annually	–41.1%	–3.74% annually	1.5× (+49%)
Trachoma	–44.3%	–1.6% annually	–23.8%	–2.2% annually	1.4× (+38%)	–59.8%	–2.2% annually	–39.1%	–3.55% annually	1.6× (+62%)

**Abbreviations:** DALY, disability-adjusted life year.

## Future directions

GBD 2016 is providing important information in order to assess the global impact of MDA and other NTD interventions, and there appear to be substantial health gains as reflected in reductions in disease burden and prevalence since 2006. With respect to disease burden assessments, de Vlas and colleagues recently estimated that NTD programs will avert some 600 million DALYs in the period 2011–2030 for ten NTDs targeted by the London Declaration [14]. Approximately 300 million DALYs averted could be attributed to the seven diseases amenable to integrated MDA, or what the WHO refers to as “preventive chemotherapy” [14]. However, as these authors point out, the calculations do not include the broader impact of NTDs on some chronic morbidities such as impaired cognitive development, the impact of stigma and discrimination and the resultant mental health burden, inappropriate health expenditures averted, and the impact on food security and agricultural productivity of afflicted individuals or communities. Future estimates may need to further consider additional aspects of the long-term disabilities, including an expanding information base of mental health effects of NTDs and the burden on caregivers of those with chronic conditions [15].

We must urgently and more effectively assess the true impact of integrated MDA and whether this approach was indeed the driver for the disease burden and prevalence reductions reported here. Implicating such associations might require individual country-level data in order to examine the effects in real time and whether they coincide with the timing of national control programs. We initially projected that the unit costs of programs would be around US$0.40 annually [1,2], and the figures from studies in Africa indicate that NTD programs based on preventive chemotherapy are indeed within that range of those costs and much lower in Asia [5, 16].

Finally, while our original call to integrate MDA also included efforts to link this approach with HIV/AIDS and malaria control [4, 9, 17], for the most part, global NTD programs have remained in silos. We are also missing important opportunities to promote other aspects of NTD control and elimination that go beyond MDA, such as vector control to drive down transmission, increasing human capacity with a knowledge base on NTDs, and clinical management of patients who continue to suffer from the long-term consequences of infection. These aspects have received less attention as the targets for MDA have been prioritized, while improved water and sanitation provision and health education need to be more vigorously encouraged.

The last decade has witnessed extraordinary progress towards global NTD control and elimination. It will be critical for this new generation of global leaders to continue their NTD commitments [18, 19].
